# Temperature-Dependent Transport of Photoinduced Charge Carriers Across a Single-Walled Carbon Nanotube Film/Si Interface

**DOI:** 10.3390/ma18194437

**Published:** 2025-09-23

**Authors:** Lizaveta A. Dronina, Aleksander L. Danilyuk, Nikolai G. Kovalchuk, Evgenii V. Lutsenko, Aleksander V. Danilchyk, Serghej L. Prischepa

**Affiliations:** 1 Laboratory “Integrated Micro- and Nanosystems”, Research and Development Department, Belarusian State University of Informatics and Radioelectronics, 220013 Minsk, Belarus; lizadronina@yandex.by (L.A.D.); n.kovalchuk@bsuir.by (N.G.K.); 2 Department of Micro- and Nanoelectronics, Belarusian State University of Informatics and Radioelectronics, 220013 Minsk, Belarus; 3 Center “Wide-Band Nano- and Microelectronics”, B.I. Stepanov Institute of Physics of NAS Belarus, 220072 Minsk, Belarus; 4 Department of Information Security, Belarusian State University of Informatics and Radioelectronics, 220013 Minsk, Belarus

**Keywords:** single-walled carbon nanotubes, Schottky barrier, thermionic emission, photocurrent, Poisson equation, photoresponsivity, photodetector

## Abstract

This study investigates the effect of temperature on the performance of the single-walled carbon nanotube (SWCNT) film/Si photodetector. Specifically, the photocurrent across a SWCNT/Si heterojunction when illuminated with light of 632.8 nm wavelength of different powers was studied in detail in a wide temperature range, from 20 to 300 K. The objective was to determine the parameters of the heterojunction, which is inherently inhomogeneous, and to identify the main ones that determine the optoelectronic figures of merit of a photodetector based on it. The barrier height and its temperature dependence were determined within the framework of the theory of thermionic emission, taking into account the non-uniform distribution of the barrier height over the heterojunction area. The parameters of the heterojunction and SWCNT/Si interface and their temperature dependences were calculated based on the known temperature dependences of the concentration of charge carriers and ionized impurities in Si using the Poisson equation based on Fermi–Dirac statistics. The obtained results indicate the importance of interplay between the effects of reducing the barrier height and the processes of decreasing the separation efficiency of nonequilibrium charge carriers and increasing the rate of their recombination.

## 1. Introduction

Nowadays, with the intensive development of photonics, significant attention is devoted to adapting the components of photonic integrated circuits for operation in extreme external environments, such as outer space, ultra-low temperatures, and high radiation levels. A key component of photonic integrated circuits that performs photoelectric conversion, the photodetector, is located at the end of the chain that converts the light signal into an electrical one, and the characteristics of the entire microcircuit ultimately rely on its performance under extreme conditions [1]. Reliable photodetector performance at low temperatures is expected to help maintain high operational parameters for the photonic integrated circuit. The influence of temperature on photoelectric conversion is closely related to nano-structuring [2]. One of the most effective nanostructures (heterostructures) for these applications is considered to be the Schottky barrier (SB), formed between a metal and a semiconductor. Efficient transport of photoinduced charge carriers across an SB is crucial for the photodetector’s operation and is known to be temperature-dependent.

In an illuminated SB, a stationary regime is attained in which the electron-hole generation rate is balanced by recombination processes, allowing the photoinduced charge carriers to relax to their steady-state distribution. With temperature variations, the mechanism of photoconductivity across the SB can change. Long-lived charge trap states caused by various interface defects and traps play a significant role in this process, reducing the photogeneration rate and photoconductivity. Additionally, noticeable changes occur in the barrier’s physical parameters with temperature. Ultimately, the extent of temperature’s influence on photoconductivity is largely determined by the materials that constitute the heterojunction. These factors highlight the importance of considering the quality of the interface, the junction parameters themselves, and the properties of the materials when describing the mechanism of photoconductivity in a heterojunction.

Heterojunctions between single-walled, metallic-conducting carbon nanotube films and silicon have attracted significant attention over the past decade [3,4,5,6,7,8]. First, Si photonics remains a mainstream technology due to substantial developments, widespread availability, and low cost [1]. Second, the single-walled carbon nanotube (SWCNT) film coating the Si acts as a conductive electrode for charge carrier collection and establishes a built-in potential for separating photocarriers [9]. Third, due to the SWCNT film’s extremely high optical transparency [10], most of the incoming light is absorbed in Si; thus, the efficiency of the SWCNT/Si device is comparable to, or even greater than, that of a conventional Si one [8]. Finally, SWCNTs are characterized by high mobility of charge carriers (>10^5^ cm^2^/V·s) [11], low internal capacitance (<10^−15^ F/µm) [12], high mechanical strength [13], and thermal stability [14]. These qualities create very favorable conditions for the development of technologically advanced, highly reliable, and promising emerging optoelectronic devices based on carbon nanotubes, as noted in a recent review by Xia et al. [15]. However, various research groups continue to seek methods to improve the performance characteristics of CNT-based photodetectors. Recent advancements include forming a voltage-active interface between SWCNT and Si [16], enhancing photoresponse by introducing copper nanoparticles into the interface between the SWCNT film and Si [17] or by applying quantum dots to the surface of the SWCNT/Si photodetector [18], as well as producing small bundles of SWCNTs on the Si surface [19].

Although significant progress has been made in developing SWCNT-based heterostructures for high-performance photodetectors [20] (see also comparison Table 1 in Ref. [8]), and the interface properties of the SB have been studied both experimentally and theoretically [21], there remains a lack of detailed information on photoinduced charge carrier transport at the metal-conducting SWCNT/Si interface, particularly at low temperatures. For example, the mechanism behind the photoelectric performance of SWCNT/Si heterojunctions has not been completely clarified due to the complexity and diversity of the SWCNT films used. Furthermore, achieving an optimal Schottky interface for SWCNTs on Si is quite challenging, as the barrier height strongly depends on the morphological features of the SWCNT film [22]. Along with the difficulty of controlling the structure and properties of SWCNTs, the lack of understanding regarding the nature of the contact between the SWCNT film and semiconductor presents a critical issue for improving and stabilizing photodetector figures of merit and integrating them into complex circuits.

In this regard, our group has recently carried out a series of studies related to the development of a reliable, simple, and reproducible technology for forming SWCNT/Si heterostructures to assess their prospects for application in optoelectronic converters. The main results of these studies are published in Refs. [8,22]. In particular, Ref. [8] describes in detail the technology for growing thin (no more than 30 nm thick) SWCNT films with predominantly metallic conductivity by floating catalyst chemical vapor deposition at atmospheric pressure directly onto a silicon substrate in the photodetector window. Detailed structural studies of the films using Raman and IR spectroscopy proved the single-walled nature of CNTs and made it possible to estimate their chirality and diameter (1.06 nm). Post-growth treatment of the SWCNT film in ethanol significantly improved the film morphology, making it denser, which led to a 20% reduction in sheet resistance. In addition, the adhesion between the film and the substrate was enhanced, which was important for significantly improving the temporal stability of the devices. The study of the dark *I*–*V* characteristics of heterojunctions within the framework of the thermionic emission theory [23] and the modified Cheung’s approach [24,25] made it possible to obtain such parameters as the height of the SB and the ideality factor. These data allowed us to conclude that the spatial inhomogeneity of the Schottky barrier, which is typical for many heterojunctions [21], was present. Based on the data obtained, the heterojunction parameters were further analyzed assuming the presence of the native tunnel oxide SiO_2_ layer between the SWCNT film and Si. Quantities such as the SWCNT work function, density of interface states, and interface charge densities were retrieved. Basic optoelectronic figures of merit such as responsivity, detectivity, and external quantum efficiency in the visible spectral range were also determined and found to be comparable to the best reported for other SWCNT-based photodetectors. All measurements in [8] were performed at room temperature.

In the following work [22], we specifically and thoroughly investigated the influence of SB inhomogeneity on the heterojunction parameters. For this reason, the dark *I*–*V* curves were measured over a wide temperature range, from 20 K to 315 K. The difference from previous studies [8] was that, in addition to using a wide temperature range, the data were elaborated not only within the framework of the thermionic approach but also by considering an analytical model adapted to thermionic emission theory over a Gaussian barrier distribution [26]. It was shown that the latter method most adequately describes the experimental data. It also follows that the entire wide temperature range can be divided into three subranges, each of which corresponds to its own barrier height value. The results showed that this factor is important for understanding the influence of the real physical structure of the barrier on the mechanisms of current transport across it.

In this work, we continue the research cycle started in works [8,22], now focusing on the photoelectric properties of the SWCNT/Si heterojunction over a wide temperature range.

Generally, the study of the photoelectric performance of SWCNT/Si heterojunctions from room to cryogenic temperatures holds significant implications. Firstly, temperature measurements enhance our understanding of the mechanisms involved in photoinduced charge transfer across the barrier. With a temperature change, parameters of materials and heterojunctions based on them, such as the density and mobility of charge carriers, energy levels, barrier height, charge density and density of states at the interface, and overall charge properties of the depletion region and the interface, can change significantly. As a result, temperature-induced changes in all these parameters create a unique opportunity to more accurately determine and refine them without affecting the object of study itself [2]. Secondly, these measurements help identify the lower temperature limit at which high-performance characteristics of photodetectors can be sustained. In this research, we measured the dark current-voltage (*I*–*V*) characteristics of the formed heterojunctions within a temperature range of 20 K to 300 K, as well as the *I–V* characteristics under illumination at various power levels and a wavelength of 632.8 nm. The dark current data were analyzed using an analytical model adapted from thermionic emission theory [23], accounting for a Gaussian distribution of the Schottky barrier height [26]. The absolute values of the barrier height at different temperatures, $\varphi_{B}(T)$, were derived within this approach. Thus, the approach described in detail in [8,22] was used for elaborating the dark *I–V* curves. However, the data presented in this paper relate to a different sample. We considered it necessary to briefly describe the method presented in [8,22], which will allow a better understanding of the essence of the work, as the analysis of the parameters extracted from the *I–V* characteristics under irradiation is largely based on the results of the temperature dependence of the barrier height extracted from the dark *I–V* curves.

Regarding the differences between our results and those of other authors, we highlight the following key points. (i) Most studies on the photodetection properties of SWCNT/Si heterojunctions use relatively low irradiation power densities, ranging from hundreds of μW/cm^2^ to tens of mW/cm^2^ [3,4,7,16,27,28]. We show that in situ formed heterojunctions operate at significantly higher power densities, on the order of hundreds of mW/cm^2^. (ii) Results are generally reported at a single temperature—room temperature. Only a few studies have examined SWCNT-based photodetectors at temperatures as low as 80–100 K [7,29,30]. To better understand the performance of photonic circuit components under extreme conditions, we investigate detector operation down to 20 K. (iii) Typically, optoelectronic figures of merit are measured and reported without a detailed analysis of the relationship between these parameters and the heterojunction properties. In this study, we measure and analyze the *I*_ph_(*T*,*P*_in_) dependencies, offering qualitative explanations based on the temperature dependence of heterojunction parameters. To accurately describe the parameters at low temperatures, the properties of the depleted Si region were calculated using the Poisson equation and the Fermi-Dirac distribution. These calculations included mobile carrier and charged donor concentrations, the electric field at the SiO_2_/Si interface, and the width of the depleted region across the entire temperature range. By applying the energy balance condition, we obtained self-consistent, temperature-dependent charge and energy properties for the SWCNT/Si heterojunction, which had not been previously achieved. This allowed us to refine interface parameters and provide a qualitative explanation of the photocurrent’s dependence on temperature and irradiation power.

The photocurrent $I_{ph}$ was determined from the reverse branch of the illuminated *I–V–T* curves, revealing its dependence on temperature $I_{ph}(T)$. Interestingly, while the barrier decreases with decreasing temperature, no corresponding increase in photocurrent was observed; instead, a steady decrease in photocurrent was observed as temperature decreased. To elucidate this discrepancy between the temperature-dependent behaviors of the barrier height and photocurrent, a comprehensive analysis of the heterojunction and interface parameters was carried out. This analysis utilized known temperature dependences of charge carrier and ionized impurity concentrations in Si, applying the Poisson equation based on Fermi-Dirac statistics. The work presents a promising approach to understanding low-temperature mechanisms of photoinduced charge carrier transport across the SWCNT film/Si SB, which may contribute to the advancement of high-performance photodetectors that operate effectively under such conditions.

## 2. Samples

**Figure 1:**
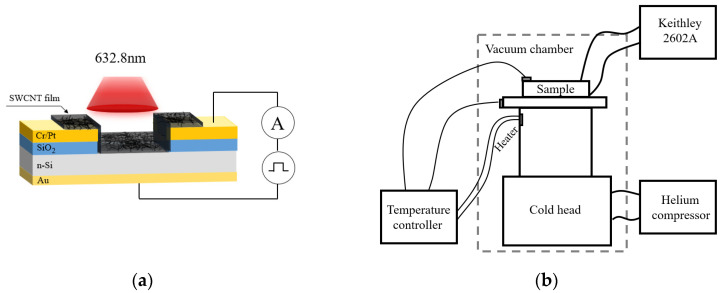
(**a**) Schematic view of the samples studied; (**b**) Block diagram of the experimental setup.

To form the Schottky barrier, 30 nm thick SWCNT films were deposited directly onto a pre-cleaned phosphorus-doped ($N_{D}$ = 10^16^ cm^−3^) Si (n-type) substrate using floating catalyst chemical vapor deposition (FCCVD) [8,31]. High-purity argon (99.99%) served as the carrier gas, while a mixture of ferrocene powder (Fe(C_5_H_5_)_2_, 98% pure) in ethanol (C_2_H_5_OH, 99.8% pure) was used as both the carbon source and catalyst. This mixture was injected at a rate of 0.2 mL/min into a quartz tube reactor (8 mm outer diameter, 1500 mm length) using a syringe-based injector for SWCNT growth. The synthesis was carried out at a temperature of 1050 °C. Afterwards, the sample was treated with ethanol (applied directly and air dried), then thermally annealed under vacuum (10^−3^ Pa) at 400 °C for 3 h. The effective area of the photodetector window was $S_{eff}$ = 0.061 cm^2^ [22]. Raman and IR spectroscopies confirmed the single-walled nature and dominant metallic conductivity of the nanotubes [8,22]. The mean nanotube diameter was approximately 1.1 nm. Figure 1a shows a schematic of the photodetector. More details about the sample’s fabrication and characterization can be found elsewhere [8,22].

A total of 8 samples were examined in this study. All were prepared under identical conditions to ensure the data are representative. The heterojunctions showed very similar properties; for example, the barrier height at room temperature varied by only a few percent. The samples were produced over several years, consistently following the same manufacturing procedures. Even after three years of storage under normal laboratory conditions and repeated thermal cycling to 20 K and back to room temperature, the “older” samples exhibited no noticeable changes in their properties. We attribute the high stability of SWCNT/Si heterojunction properties mainly to post-deposition treatment, which leads to film compaction and better contact with the silicon surface [22]. This paper presents data for a sample that has not been previously presented.

For the electrical measurements at different temperatures, samples were placed inside an optical helium closed-cycle cryostat Janis CCS-150 (Wilmington, MA, USA) equipped with a ZnSe window. The temperature varied within the range of 20–300 K. The experimental setup schematically shown in Figure 1b. The copper holder served as a platform for mounting the sample, and silver paste was used to create an ohmic contact with the back of the silicon substrate. Additionally, the connection to the upper electrodes was achieved using thin silver-plated small gauge wires. Once the sample mounted to the cold head, the radiation shield and vacuum shroud were installed. Measurements were conducted in vacuum at a pressure lower than 5 × 10^−6^ mbar and the temperature was monitored using an automatic temperature controller, silicon diode thermometer, and 25 Ohm control heater. One of the reference sensors was placed in thermal contact with the sample under the measurements. The thermal stability of the overall equipment was monitored by using a second sensor, positioned on the sample stage. Temperature controller was used to automatically control the temperature set-points. Measurements on our sample were performed after waiting for the settling of stable and equal temperatures measured on both sensors for at least 10 min at each *T*. The temperature stabilization during each acquisition was better than 50 mK. Both forward and reverse branches of *I*–*V* characteristics were registered at each temperature with steps of 10 mV in the range of −3 V to +3 V. Photo response measurements were conducted in photovoltaic mode using a Keithley 2602A source–meter (Clivelend, OH, USA) and a He-Ne laser with a wavelength of λ = 632.8 nm, which may vary radiation intensity through an optical attenuator. The laser beam spot with a diameter of 4 mm was centered on the SWCNT/Si window. Light intensity $P_{in}$ was measured with a thermal power sensor PM100D, supplied by Thorlabs Inc. (Newton, NJ, USA), and positioned at the sample’s location. The range of $P_{in}$ was varied from 8.2 to 268.8 mW/cm^2^. The photocurrent $I_{ph}$ (as well as dark current $I_{d}$) was determined from the backward branch of the *I*–*V* characteristic at a voltage of −2 V. From the forward *I*–*V* curves, the Schottky barrier height $\varphi_{B}$ was extracted.

## 3. Results

**Figure 2:**
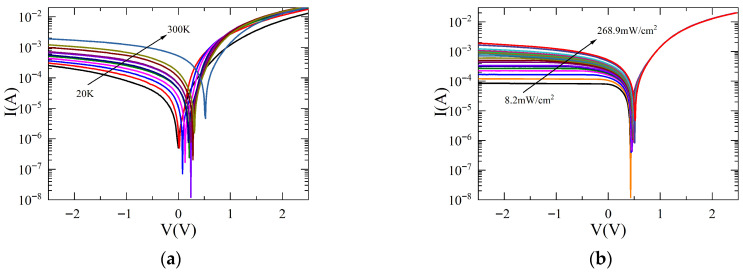
(**a**) *I*–*V* characteristics at different *T*, $P_{in}=$ 268.9 mW/cm^2^. Different colors correspond to different temperatures; (**b**) *I*–*V* characteristics at different $P_{in}$, *T =* 300 K. Different colors correspond to different powers.

Typical *I–V* characteristics of the SWCNT/Si heterojunction, taken at different temperatures and fixed $P_{in}=$ 268.9 mW/cm^2^, are shown in Figure 2a. Figure 2b shows the *I–V* characteristics taken at a fixed temperature (*T* = 300 K), but at different $P_{in}$.

**Figure 3:**
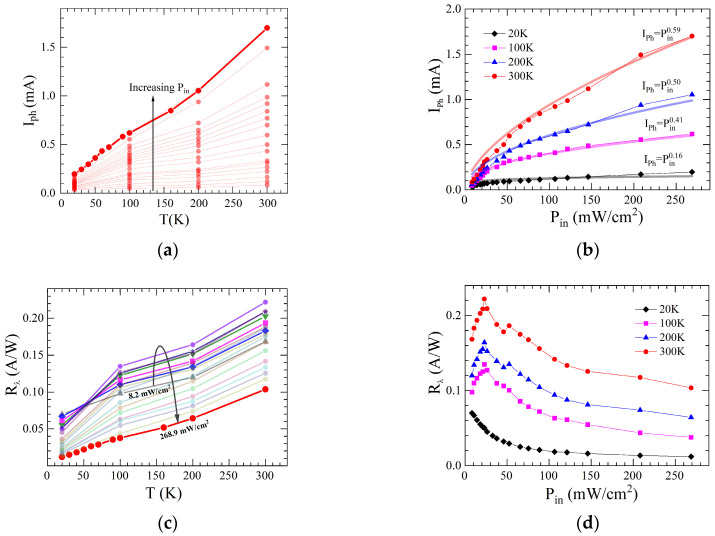
(**a**) Photocurrent as a function of *T* at various $P_{in}$; (**b**) Photocurrent versus $P_{in}$ at different *T* (symbols). The solid lines are for the best power fits to the experimental data. Inset: Exponent α versus *T*; (**c**) Responsivity versus *T* at different $P_{in}$. Different colors correspond to different powers. The arrow indicates the direction of power increase; (**d**) Responsivity versus $P_{in}$ at different *T*.

In Figure 3a, we plot $I_{ph}(T)$ dependencies measured at different $P_{in}$. It follows that the photocurrent decreases as the temperature decreases. Notably, as radiation power decreases, the changes in current with temperature become less steep. At the maximum radiation power we use, 268.8 mW/cm^2^, the photocurrent decreases by an order of magnitude with a change in temperature from 300 K to 20 K; at the minimum power, 8.2 mW/cm^2^, it decreases by only a factor of two.

The relationship between laser power density and photocurrent was further investigated to reveal the response characteristics of the SWCNT/Si heterojunction. When the power increases from 8.2 to 268.8 mW/cm^2^, the photocurrent at *T* = 300 K increases up to 1.7 mA. The relationship between the two variables for different temperatures has been summarized in Figure 3b. The obtained dependencies can be well fitted by the power law, $I_{ph}\propto P_{in}^{\alpha }$ with the exponent α<1. In general, the photocurrent is linearly proportional to the light power in the absence of charge trapping in the heterojunction region. In contrast, long-lived charge traps of various origins result in a deviation from the linear relationship with the exponent α<1. A detailed analysis of the temperature dependence of the exponent α reveals that it decreases with decreasing temperature, from 0.59 at *T* = 300 K to 0.16 at *T* = 20 K. This result is shown in Figure 3b. This behavior of the exponent α indicates an increase in the capture of photoinduced charge carriers through the SB at low temperatures.

Responsivity $R_{\lambda }$ is a significant parameter to evaluate the photodetector sensitivity $R_{\lambda }=(I_{ph}-I_{d})/(P_{in}\times S_{eff})$. Note that the dark current in our case did not exceed 0.5 µA over the entire temperature range. The $R_{\lambda }(T)$ dependencies at different powers are shown in Figure 3c. In general, a decrease in photoresponsivity is observed with decreasing temperature for all light intensities. However, if for high light intensities ($P_{in}$ > 200 mW/cm^2^) the decrease in $R_{\lambda }$ occurs by an order of magnitude, then for low powers ($P_{in}$ < 10 mW/cm^2^) it is only 2 times. Therefore, this experimental result demonstrates that at low illumination powers, the photoresponsivity of the SWCNT-based photodetector at low temperatures is quite stable and varies slightly over a wide temperature range.

In Figure 3d, we show the responsivity as a function of $P_{in}$ for different temperatures. The noticeable behavior of the $R_{\lambda }(P_{in})$ dependence at low powers is noteworthy. Up to $P_{in}$ = 22.95 mW/cm^2^, responsivity at *T* > 20 K increases quite quickly, and at higher illumination powers, it begins to gradually decrease. On the other hand, at *T* = 20 K, the $R_{\lambda }(P_{in})$ dependence is a monotonic, smoothly decreasing function with a tendency to saturate at $P_{in}>$ 70 mW/cm^2^. This result indicates that at low temperatures the transport of photoinduced charge carriers across the SB is not effective at any value of $P_{in}$, while at *T* ≥ 100 K, at first, with an increase in the illumination power, the generation of photoinduced charge carriers increases, and then, after exceeding the threshold power, recombination of these carriers occurs.

## 4. Discussion

### 4.1. Temperature Dependence of the SB Height

We begin our discussion of the obtained data with an analysis of the temperature behavior of the SB height. To achieve this, we apply several approaches, such as thermionic emission (TE) theory [23] modified by a simple correction to the ideality factor [25], and an analytical model adapted to the TE theory over a Gaussian barrier distribution [26]. In the modified TE approach, the forward-bias *I–V* characteristic is expressed as [25]

(1) $$I=S_{eff}A^{*}T^{2}e^{-\sqrt{\chi }\delta }e^{-\frac{\varphi_{B}^{TE}}{kT}}\left[e^{\frac{q(V-IR_{S})}{\eta kT}}-1\right],$$

where $\varphi_{B}^{TE}$ is the SB height determined within the TE approach, η is the ideality factor, $A^{*}$ is the Richardson constant, which, for *n*-Si, is assumed to be approximately 112 A/cm^2^K^2^, $R_{S}$ is the series resistance of the SB, *k* stands for the Boltzmann constant, q represents the magnitude of electronic charge, and χ (in eV) is the mean tunneling barrier height. Additionally, δ (in Å) is the interface oxide thickness, which is assumed to be 3 nm [8].

**Figure 4:**
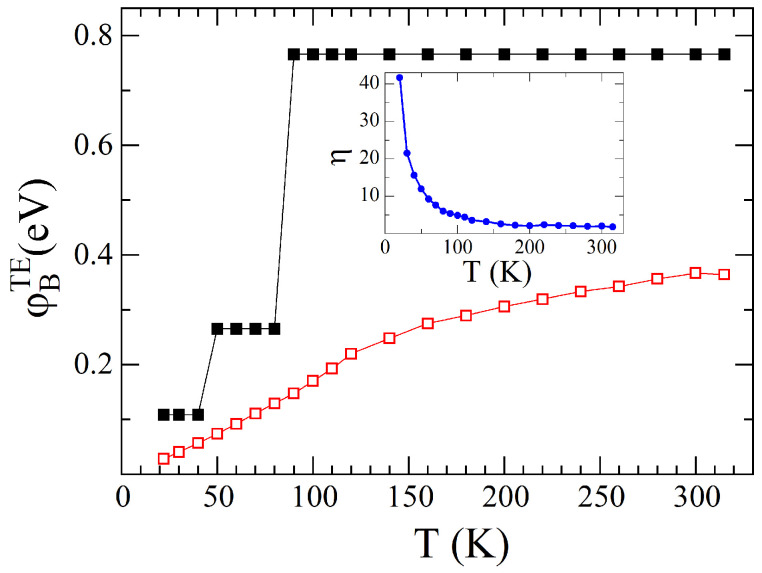
Temperature dependence of the $\varphi_{B}^{TE}$ (open squares) and ${\bar{\varphi }}_{B}$ (closed squares). Inset: the ideality factor versus temperature.

Based on Equation (1), one can extract the barrier height and the ideality factor and plot their temperature dependences. The result obtained for the barrier height is shown in Figure 4, and the η(T) dependence is present in the inset. The barrier decreases with decreasing temperature, whereas the ideality factor increases. Although the TE theory is commonly used to obtain SB parameters, deviations from classical TE theory occur at low temperatures regardless of the materials on which the Schottky contact is formed [22,26,30,32,33]. Such temperature dependence of the SB parameters indicates that spatial inhomogeneities are effective at the heterojunction interface [26,34,35]. The increase in the ideality factor greatly beyond 1 implies nonideal transport across the SB. Considering the temperature-activated process, at low temperatures, small SBs are involved in carrier transport. At high temperatures, these low barriers are masked by barriers with greater amplitude, thus increasing the effective measurable barrier height. In addition, current transport becomes more spatially uniform. All of these factors lead to a decrease in the ideality factor and an increase in the SB height at high temperatures. Such non-uniformities in barrier height can be accounted for by introducing a statistical Gaussian distribution of the barrier height [26]. This Gaussian thermionic approach can be successfully applied to describe the properties of SBs over a wide temperature range. However, taking into account the fact that the actual distribution of the barrier height can be quite complex, due to which it is impossible to describe the experimental dependence of the barrier height on temperature with one set of parameters, it is customary to divide the temperature range into several, two or three, sub-ranges, each with its distribution parameters [22,33]. For heterojunctions based on SWCNTs, such an approach is even more relevant given the non-uniform contact of the nanotubes with the semiconductor surface [22].

The essence of this method is that the barrier is assigned a mean value ${\bar{\varphi }}_{B}$ and standard deviation from the mean $\sigma_{B}$. Then the total current is expressed as

(2) $$J\left(\varphi_{ap}\right)=J_{0}\left(e^{\frac{qV}{kT}}-1\right),$$

where $J_{0}=A^{*}T^{2}e^{-\frac{\varphi_{ap}}{kT}}$ and the apparent barrier height $\varphi_{ap}$ is introduced [36]

(3) $$\varphi_{ap}={\bar{\varphi }}_{B}-\frac{\sigma_{B}^{2}}{2kT}+kTln\left[1+erf\left(\frac{{\bar{\varphi }}_{B}}{\sqrt{2}\sigma_{B}}\right)\right]-kTln\left[1+erf\left(\frac{{\bar{\varphi }}_{B}-\frac{\sigma_{B}^{2}}{kT}}{\sqrt{2}\sigma_{B}}\right)\right],$$

where erf denotes the error function.

Analyzing the *I*–*V*–*T* characteristics within the framework of such an approach, we find that they can be described through the following relationship:

(4) $$R\equiv \left(\frac{\sigma_{B}^{2}}{{2k}^{2}T^{2}}\right)=ln\left(\frac{J_{0}}{T^{2}A_{eff}^{*}}\right)+\frac{{\bar{\varphi }}_{B}\;}{kT}.$$

In this case, as was demonstrated in [22], the entire studied temperature range is divided into three sub-ranges: 315–90 K, 80–50 K, and 40–20 K. At each of these sub-ranges the SB is characterized by its value of ${\bar{\varphi }}_{B}$. Thus, depending on the elaboration procedure, both $\varphi_{B}^{TE}$ and ${\bar{\varphi }}_{B}$ values at different temperatures can be extracted from the forward-bias *I*–*V* curves. Their values for different temperature sub-ranges are summarized in Table 1. In Figure 4, the closed symbols demonstrate the ${\bar{\varphi }}_{B}(T)$ dependence. For a more detailed description of the elaboration procedure of the *I*–*V*–*T* characteristics by the discussed two methods, readers are referred to our recent publication [22].

Figure 4 shows that, regardless of the method used to elaborate the *I*–*V*–*T* characteristics, the barrier height decreases as the temperature goes down. At first glance, this seems to contradict the data on how photocurrent depends on temperature. Since the barrier decreases with decreasing temperature, one might expect the photocurrent to increase. But since this is not the case, a more detailed analysis of the heterojunction parameters and their temperature changes is necessary.

### 4.2. Main Parameters of the SWCNT/Si Heterojunction and Their Temperature Dependencies

**Figure 5:**
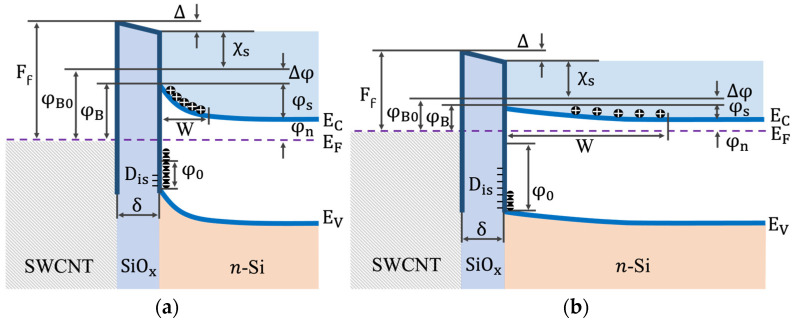
Energy-band diagram of the SWCNT/Si heterojunction with its main parameters. (**a**) *T* = 300 K; (**b**) *T* = 20 K. For explanation see the text.

The primary parameters of the heterojunction that need to be analyzed are defined by its energy-band diagram, shown in Figure 5. The following notations are used. $\varphi_{B}$ is the SB height at a given temperature (here we neglect the Gaussian distribution of the SB height), $\varphi_{B0}$ is the SB height at zero electric field, $F_{f}$ represents the work function of the SWCNT film, $\varphi_{0}$ denotes the charge neutrality level (CNL), ∆φ indicates the barrier lowering due to the image forces, Δ signifies the potential drop on the intermediate oxide layer, $\chi_{S}$ stands for the electron affinity of Si, $\varphi_{n}=E_{C}-E_{F}=(kT/q)\ln\left(N_{D}/N_{C}\right)$ is the difference between the Fermi level *E_F_* and the conduction band minimum $E_{C}$, $E_{V}$ represents the valence band maximum, and $\varphi_{S}=\varphi_{B}-\varphi_{n}$ is the band bending in the Si depleted layer. Additionally, *N_C_* refers to the effective density of states in the conduction band, while *N_D_* indicates the concentration of donors. In Figure 5a, the energy-band diagram for *T* = 300 K is present, and in Figure 5b, the corresponding diagram for *T* = 20 K is shown. The difference in the energy diagrams related to different temperatures follows from the temperature dependences of the heterojunction parameters, which will be discussed below.

We start with the calculation of the $\varphi_{n}(T)$ dependence by applying the condition of quasi-neutrality in the silicon volume [37],

(5) $$p+N_{D}^{+}-n=0,\;$$

where p, n, $N_{D}^{+}$ are the concentrations of holes, electrons, and positively charged donors in the quasi-neutral region of silicon, respectively. They are determined according to the known expressions [37]:

(6) $$n=N_{C}\frac{2}{\sqrt{\pi }}F_{1/2}\left(\frac{E_{F}-E_{C}}{kT}\right),$$

(7) $$p=N_{V}\frac{2}{\sqrt{\pi }}F_{1/2}\left(\frac{E_{V}-E_{F}}{kT}\right),$$

(8) $$N_{D}^{+}=N_{D}\left[1-\frac{1}{1+\frac{1}{g}\exp\left(\frac{E_{D}-E_{F}}{kT}\right)}\right].$$

**Figure 6:**
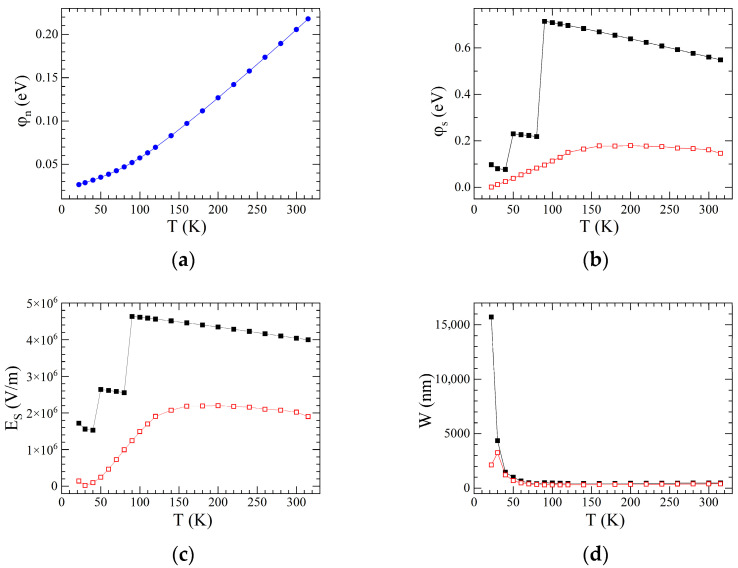
(**a**) **$\varphi_{n}$** versus *T* for SWCNT/Si heterojunction; (**b**) $\varphi_{S}$ versus *T* for SWCNT/Si heterojunction; (**c**) *E*_S_ versus *T* for SWCNT/Si heterojunction; (**d**) *W* versus *T* for SWCNT/Si heterojunction. Open (closed) symbols are for $\varphi_{B}^{TE}({\bar{\varphi }}_{B}$) values.

In Equations (6)–(8) $N_{V}$ is the effective density of states in the valence band, $F_{1/2}$ is the Fermi–Dirac integral, g=2 is the degeneracy factor of the donor impurity level, and $E_{D}$ is the ionization energy of the donor level. For phosphorus $E_{D}=$ 45 meV [37]. The obtained $\varphi_{n}(T)$ dependence is shown in Figure 6a. With decreasing temperature, the Fermi level approaches the bottom of the conduction band. This behavior is typical for doped n-type semiconductors.

Knowing how $\varphi_{n}$ and $\varphi_{B}$ change with temperature, we obtain the change in $\varphi_{S}$. This result is shown in Figure 6b. Calculations were carried out for two $\varphi_{B}(T)$ dependencies, both for $\varphi_{B}^{TE}$ and ${\bar{\varphi }}_{B}$. As follows from Figure 6b, the “rates” of temperature changes of $\varphi_{n}$ and $\varphi_{B}$ are such that, overall, $\varphi_{S}$ decreases with temperature. This means that the bending of the conduction band bottom decreases as temperature lowers. This, in turn, should lead to a decrease in the built-in potential of the heterojunction.

To check this important statement, we further calculate the field strength $E_{S}$ at the Si/SiO_2_ interface. Calculations were performed applying Poisson’s equation for the electrostatic potential ϕ(x) in the depleted Si layer,

(9) $$\frac{d^{2}\phi (x)}{dx^{2}}=\frac{q}{\varepsilon_{S}\varepsilon_{0}}\left[p\left(\phi \right)+N_{D}^{+}-n(\phi )\right],$$

where *x* is the coordinate normal to the Si/SiO_2_ interface, $\varepsilon_{0}$ is the permittivity of vacuum, $\varepsilon_{S}=\;11.8$ and $\varepsilon_{i}=$ 3.9 are the relative permittivity of Si and SiO_x_, respectively, $p\left(\phi \right)$ and n(ϕ) are the holes and electrons concentrations, respectively, in the depleted layer, determined as [37]

(10) $$n(\phi )=N_{C}\frac{2}{\sqrt{\pi }}F_{1/2}\left(\frac{E_{F}-E_{C}+q\phi (x)}{kT}\right),$$

(11) $$p(\phi )=N_{V}\frac{2}{\sqrt{\pi }}F_{1/2}\left(\frac{E_{V}-E_{F}-q\phi (x)}{kT}\right).$$

Substituting Equations (10) and (11) into Equation (9) and integrating over ϕ, one obtains the expression for $E_{S}$,

(12) $$E_{S}={\left(\frac{2q}{\varepsilon_{S}\varepsilon_{0}}\right)}^{1/2}{\left[\int_{0}^{\varphi_{S}}\left[p\left(\phi \right)+N_{D}^{+}-n(\phi )\right]d\phi +C\right]}^{1/2},$$

where C is the integration constant. The $E_{S}(T)$ dependencies for both $\varphi_{B}^{TE}$ and ${\bar{\varphi }}_{B}$ is shown in Figure 6c. Obviously, the electric field decreases with decreasing temperature.

Based on the Poisson’s Equation (9) it is possible to arrive at the expression for the depleted layer width *W*. For that, we integrated Equation (9) twice,

(13) $$W={\left(\frac{\varepsilon_{S}\varepsilon_{0}}{2q}\right)}^{1/2}\int_{0}^{\varphi_{S}}\frac{d\phi }{{\left[\int_{0}^{\varphi_{S}}\left[p\left(\phi \right)+N_{D}^{+}-n(\phi )\right]d\phi +C\right]}^{1/2}}.$$

The obtained W(T) dependencies are plotted in Figure 6d for both $\varphi_{B}^{TE}$ and ${\bar{\varphi }}_{B}$.

Further, the relations between parameters of the heterojunction SWCNT/SiO_x_/Si within the band diagram presented in Figure 5 can be set according to the expression [23,37],

(14) $$F_{f}-\chi_{s}-\varphi_{B}-\Delta \varphi =\sqrt{\frac{2q^{2}\varepsilon_{s}\varepsilon_{0}N_{D}\delta^{2}\left(\varphi_{B}+\Delta \varphi -\varphi_{n}-kT\right)}{{\left(\varepsilon_{i}\varepsilon_{0}\right)}^{2}}}-\frac{q^{2}D_{is}\delta }{\left(\varepsilon_{i}\varepsilon_{0}\right)}\left(E_{g}-\varphi_{0}-\varphi_{B}-\Delta \varphi \right),$$

where *E_g_* is the silicon energy gap, *D_is_* is the density of interfacial states on the SiO_x_/Si interface, thickness of native SiO_x_ layer δ= 3 nm [8]. Note that within the accepted models, it is assumed that *D_is_* = *const* in the energy range from $\varphi_{0}$ to *E*_F_.

**Figure 7:**
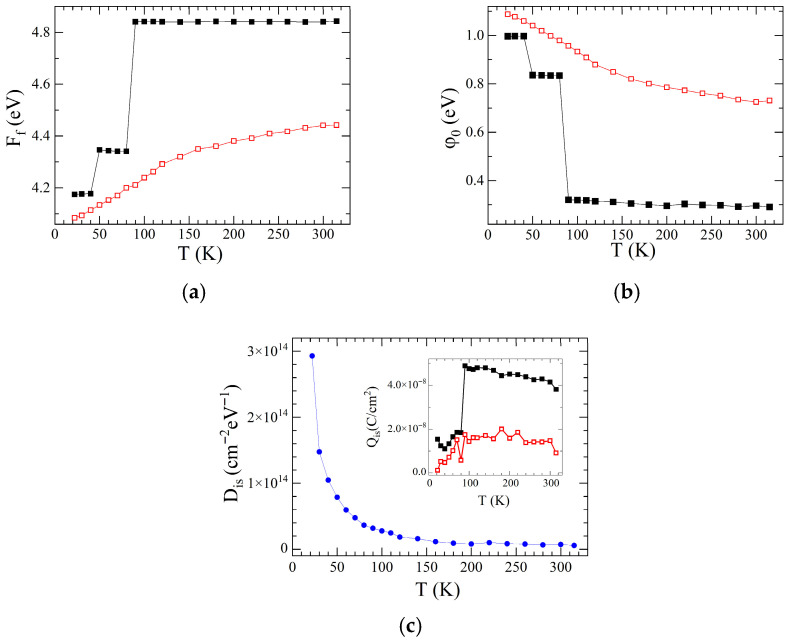
(**a**) Variations in the work function $F_{f}$ with temperature; (**b**) Variations in the CNL with temperature; (**c**) $D_{is}$ versus temperature Inset: $Q_{is}$ versus temperature. Open (closed) symbols are for $\varphi_{B}^{TE}({\bar{\varphi }}_{B}$) values.

Based on the experimental data obtained and preliminary estimates, the parameters of the SB were calculated by varying the quantities contained in Equation (14). The variation was carried out following the inequalities limiting the allowable ratios between the parameters and the regions of their change: $F_{f}-\chi_{S}-\varphi_{B}-\Delta \varphi >0$, $E_{g}-\varphi_{0}-\varphi_{B}-\Delta \varphi >0.\;$ As a result, such parameters as $F_{f}$ (Figure 7a), $\varphi_{0}$ (Figure 7b), Δ, were calculated, and the values of $D_{is}$ (Figure 7c) and $\Delta \varphi =\sqrt{qE/\left(4\pi \varepsilon_{s}\varepsilon_{0}\right)}$ were refined. Using the obtained values, the surface charge densities on the SiO_x_/Si interface, $Q_{is}=-qD_{is}\left(E_{g}-\varphi_{0}-\varphi_{B}-∆\varphi \right)$ was further calculated, see inset to Figure 7c.

### 4.3. The Temperature Dependence of the Photocurrent Across the SWCNT/Si Heterojunction

The measured temperature dependences of the photocurrent should be analyzed both from the perspective of changes in the parameters of the heterojunction with temperature, and the absorption properties of bulk silicon. First, the obtained SB parameters have significantly helped us shed light to a large extend on the observed temperature and power dependencies of the photocurrent. Along with the decrease in the barrier height with decreasing temperature, there is also a decrease in the work function $F_{f}$ of the SWCNT array, in φ_n_, φ_S_, and *E*_S_, and an increase in φ_0_, *W*, and *D_is_*. Note that the growth of *D_is_* is associated with an increase in both the non-ideality factor and the extension of the depleted Si layer. Decreasing the SB height, while keeping other parameters equal, should lead to an increase in the photocurrent, which is not observed. This is counteracted by effects such as a decrease in the potential φ_S_, leading to a decrease in the field strength *E*_S_, as well as an increase in the extension of the depleted layer *W*. The decrease in the SB height and *E*_S_ contributes to a decrease in the separation efficiency generated by the emission of nonequilibrium electrons and holes in the SB region. An increase in the CNL leads to a decrease in charge on the SiO_x_/Si interface *Q*_is_ with decreasing temperature, despite an increase in the density of states *D_is_* and a decrease in SB height. An increase in *D_is_* along with a decrease in *Q*_is_ leads to an increase in the surface recombination rate of the generated electrons and holes. An increase in *W* leads to an expansion of the bulk recombination region. It can also be assumed that, with a decrease in temperature in the SWCNT array the processes of neutralization of holes by means of trap states are activated. The increase in both interface and bulk recombination processes at low temperatures also reduces the influence of irradiation power on the magnitude of the photocurrent.

We will not disregard the fact that the reduction in photocurrent could also be affected by a decrease in the absorption coefficient of Si at low temperatures [38,39,40]. Assessments of the absorption coefficient, based on the complex refractive index for single-crystal silicon [41], reveal that for a wavelength of 632 nm it decreases by 1.672 times with a decrease in temperature from 300 to 20 K. This may also contribute to a certain extent, to the decrease in photocurrent due to the smaller number of charge carriers generated by light.

Thus, the decrease in photocurrent with decreasing temperature results from a competition between two main factors: on one hand, the reduction in the SB height and the processes that decrease the separation efficiency of nonequilibrium charge carriers, as well as the increased rate of their recombination; and on the other hand, the reduction in the number of photoinduced charge carriers.

The separation efficiency can be improved, and the recombination rate at low temperatures can be decreased by adjusting the electronic properties of SWCNTs. For example, enhancing the hole transport mechanism—similar to what happens in heterojunction solar cells with metal oxides [42]—can be effective. This mechanism in SWCNTs may be achieved by doping with impurities that create localized states within the band gap, allowing holes to tunnel between them [42]. Additionally, doping SWCNTs with boron can generate holes in the valence band [43], which helps improve hole extraction efficiency from Si through the barrier and consequently reduces the recombination rate. Other known methods for modifying the electronic structure of SWCNTs [44], along with band engineering and heterojunction interface tuning techniques used in other materials [45], can further support these improvements.

## 5. Conclusions

In this work, we thoroughly investigated the influence of temperature on the optoelectronic performance of SWCNT film/Si photodetectors. Experimental data revealed that photocurrent decreases with a decrease in temperature and increases with the growth of $P_{in}$. At low light intensities, changes in temperature and light power have less impact. To interpret these findings, we examined heterojunction parameters and their temperature dependencies. Whereas decreasing temperature lowers the Schottky barrier height, it also reduces band bending in the Si depleted layer, which decreases the built-in potential and the charge separation rate. The Si/SiO_2_ interface is also significant. *D_is_* and *Q_is_* behave differently with temperature, increasing interface recombination at low temperatures and, along with a rise in *W*, expanding the recombination region. In summary, our work clarifies the roles that SWCNT/Si heterojunction parameters play in device performance, providing a theoretical and experimental framework for their optimization.

## Figures and Tables

**Table 1:** Temperature-dependent SB heights of SWCNT/Si heterojunctions obtained by different approaches.

Temperature Sub-Range, K	$\varphi_{B}^{TE}$, eV	${\bar{\varphi }}_{B}$, eV
90–315	0.147–0.364	0.766
50–80	0.074–0.129	0.265
20–40	0.025–0.057	0.109

## Data Availability

The original contributions presented in this study are included in the article. Further inquiries can be directed to the corresponding author.
